# Olive phenols preserve lamin B1 expression reducing cGAS/STING/NFκB‐mediated SASP in ionizing radiation‐induced senescence

**DOI:** 10.1111/jcmm.17255

**Published:** 2022-03-12

**Authors:** Elena Frediani, Francesca Scavone, Anna Laurenzana, Anastasia Chillà, Katia Tortora, Ilaria Cimmino, Manuela Leri, Monica Bucciantini, Monica Mangoni, Gabriella Fibbi, Mario Del Rosso, Alessandra Mocali, Lisa Giovannelli, Francesca Margheri

**Affiliations:** ^1^ Department of Experimental and Clinical Biomedical Sciences University of Florence Florence Italy; ^2^ Department of Neurofarba (Department of Neurosciences, Psychology, Drug Research and Child Health) University of Florence Florence Italy; ^3^ AOU Meyer Florence Italy; ^4^ Radiation Oncology Unit ‐ Oncology Department Azienda Ospedaliero Universitaria Careggi Florence Italy; ^5^ Department of Translational Medicine Research Unit (URT) Genomic of Diabetes Institute of Experimental Endocrinology and Oncology National Council of Research (CNR) University of Naples Federico II Naples Italy

**Keywords:** DNA damage, human fibroblasts, polyphenols, radiation‐induced senescence, SASP

## Abstract

Senescence occurs upon critical telomere shortening, or following DNA damage, oncogenic activation, hypoxia and oxidative stress, overall referred to stress‐induced premature senescence (SIPS). In response to DNA damage, senescent cells release cytoplasmic chromatin fragments (CCFs), and express an altered secretome, the senescence‐associated secretory phenotype (SASP), which contributes to generate a pro‐inflammatory and pro‐tumoral extracellular milieu. Polyphenols have gained significant attention owing to their anti‐inflammatory and anti‐tumour activities. Here, we studied the effect of oleuropein aglycone (OLE) and hydroxytyrosol (HT) on DNA damage, CCF appearance and SASP in a model of irradiation‐induced senescence. Neonatal human dermal fibroblasts (NHDFs) were γ‐irradiated and incubated with OLE, 5 µM and HT, 1 µM. Cell growth and senescence‐associated (SA)‐β‐Gal‐staining were used as senescence markers. DNA damage was evaluated by Comet assay, lamin B1 expression, release of CCFs, cyclic GMP‐AMP Synthase (cGAS) activation. IL‐6, IL‐8, MCP‐1 and RANTES were measured by ELISA assay. Our results showed that OLE and HT exerted a protective effect on 8 Gy irradiation‐induced senescence, preserving lamin B1 expression and reducing cGAS/STING/NFκB‐mediated SASP. The ability of OLE and HT to mitigate DNA damage, senescence status and the related SASP in normal cells can be exploited to improve the efficacy and safety of cancer radiotherapy.

## 1 INTRODUCTION

Cellular senescence is an irreversible permanent cell cycle arrest accompanied by changes in cell morphology and physiology.^1^ Depending on the activating signals it can be categorized as replicative and premature senescence.^2^ Replicative senescence was first described by Hayflick and Moorhead in 1961 in normal mammalian cells characterized by a finite replicative potential,^3^ currently known to be induced via signals triggered by telomere shortening.^4^ Stress‐Induced Premature Senescence (SIPS), on the other hand, can be induced in young cells by various stressors such as DNA damaging agents, oxidative stress, mitochondrial dysfunction and others.^5^, ^6^, ^7^, ^8^

Among the typical hallmarks of senescence are enlarged and flattened morphology, a less regular shape, a larger nucleus,^5^ increased activity of lysosomal senescence‐associated β‐galactosidase, (SA)‐β‐Gal,^9^ and increase in tri‐methylated lysine 9 of histone H3 (H3K9‐3me) reflecting the formation of senescence‐associated heterochromatin foci (SAHF).^10^, ^11^ In addition, DNA damage response is also known as a crucial mediator of cellular senescence and the phosphorylation of the histone variant γ‐H2AX is another widely used marker of cellular senescence.^12^ Increased DNA leakage through the nuclear membrane has also been reported in senescence, possibly associated to the downregulation of the nuclear lamina protein lamin B1, also used as a marker for senescent cells.^13^ Dou et al. showed that cytoplasmic chromatin deriving from this leakage plays a crucial role in promoting the pro‐inflammatory senescence‐associated secretory phenotype (SASP) in human fibroblasts, activating an immune response to cytoplasmic chromatin fragments (CCFs), the cGAS‐STING pathway. In addition, they demonstrated that the silencing of cGAS‐STING pathway was effective in reducing NFκ B‐mediated SASP programme, with lower effect on γ‐H2AX, senescence‐associated heterochromatin foci or CCFs in senescent cells.^14^

Oleuropein is the most abundant polyphenol in the leaves and fruit of the olive plant and is a potent antioxidant agent with anti‐tumour and anti‐inflammatory properties.^15^, ^16^ The mechanism of the anti‐tumour effect of oleuropein and other olive secoiridoids is probably complex, involving among the others induction of apoptosis and autophagy modulation.^16^ The major oleuropein metabolite, hydroxytyrosol has also been reported to exert anti‐tumour activities through the modulation of different signalling and metabolic pathways.^16^

We have previously reported that the chronic treatment with low doses of oleuropein aglycone and hydroxytyrosol can reduce NFkB activation and the secretion of pro‐inflammatory SASP factors during the development of senescence.^17^, ^18^ Senescence‐associated secretory phenotype is considered an important factor in the development of a cancer‐favouring microenvironment, and the modulation of inflammatory signalling in senescence can be one of the mechanisms by which these olive phenols exert anti‐cancer activity. In fact, we have recently reported that the pro‐angiogenic effect of senescent fibroblast‐conditioned media on progenitor endothelial cells was reduced after fibroblast pretreatment with oleuropein aglycone.^18^ Similar results were also found for resveratrol^19^, ^20^ for which protective effects on senescence‐associated DNA damage were also reported.^21^ We thus hypothesized that an increased nuclear DNA stability might lead to reduced DNA leakage in the cytoplasm and, in turn, to reduced inflammatory signalling from cytoplasmic DNA‐sensing pathways.

To evaluate this hypothesis, we analysed the effect of oleuropein aglycone and hydroxytyrosol on DNA damage, CCF appearance and associated intracellular signalling, and SASP in a model of irradiation‐induced senescence in neonatal human dermal fibroblast (NHDFs).

## 2 METHODS

### 2.1 Olive phenolic compounds

Hydroxytyrosol (3,4‐dihydroxy‐phenylethanol) was obtained from SIGMA Cayman Chemicals (Vinci Biochem, Vinci, Italy), and oleuropein from Extrasynthese (Lyon, France). To obtain the corresponding aglycone, oleuropein was subjected to a de‐glycosilation procedure according to.^22^ The aglycone obtained was referred to as OLE throughout the paper. Hydroxytyrosol (HT) was dissolved in water (100 mM) and oleuropein in dimethyl sulfoxide (DMSO) (50 mM). Both solutions were then serially diluted in culture media to obtain the final concentration, with the percentage of ethanol and DMSO in the medium being below 0.1%.

### 2.2 Cell culture

Neonatal human dermal fibroblasts (NHDFs) were obtained from Lonza (Euroclone, Pero, Italy). The NHDFs were cultured in high‐glucose (4500 g/L) Dulbecco's Modified Eagle Medium (DMEM), supplemented with 10% foetal bovine serum, 2 mM L‐glutamine, 100 units/mL penicillin and 100 μg/mL streptomycin (Sigma‐Aldrich, Milan, Italy) at 37°C in 5% CO2 humidified atmosphere. At confluence, cultures were propagated by trypsinization, and the attained population doubling level (PDL) was calculated for NHDFs according to the equation: PDL = 3.32 × logN/N0 (where N and N0 are the recovered and seeded cell numbers respectively).

### 2.3 Induction of senescence by ionizing radiation (IR) and polyphenol treatment of NHDFs

Pre‐senescent NHDF cultures (contained >70% proliferating cells and were <10% senescence‐associated βgalactosidase positive cells) were seeded, grown to confluence, and then irradiated with γ‐radiation delivered at 146 Gy/h with a 137Cs source by using a GammaCell 1000 irradiator. Single doses of 6, 8 and 10 Gy were delivered to the cell cultures. Pre‐senescent cells mock‐irradiated (placed inside the irradiator for the same interval used for the irradiated samples, but without exposure to radiation) were used as a control. 24 h post irradiation, the irradiated and mock‐irradiated cell cultures were treated continuously with 5 µM oleuropein (OLE) or 1 µM hydroxytyrosol (HT), for 2 weeks (5 treatments) as previously described.^19^ The HT and OLE dosage choice is based on preliminary experiments performed with different doses: 5 and 1 µM represent the minimum effective dose for OLE and HT respectively.

The culture medium was replaced every 2 days to maintain the concentration of the phenolic compounds relatively constant over time.

### 2.4 Proliferation assay

The treatments with OLE and HT were performed for 2 weeks. At the end of treatment, the cells were recovered, partly seeded in T25 flasks (150.000 cells/T25) and counted after 24, 48 and 72 h.

### 2.5 (SA)‐β‐Gal Assay

After irradiation, for the assessment of senescence‐associated (SA)‐β‐Gal activity, cells were seeded at a density of 10–20 cells cm^2^ and then, treated continuously with 5 µM OLE or 1 µM HT for 2 weeks (5 treatments). (SA)‐β‐Gal‐staining was performed according to Dimri et al.^9^ Cells were washed once with phosphate buffered saline (PBS), fixed with 3.7% formaldehyde and rinsed three times with water. Thereafter, cells were incubated with staining buffer [1 mg/ml−1 X‐Gal (5‐bromo‐4‐chloro‐3‐indolyl β‐d‐galactoside), 5 mM K3Fe [CN]6,5 mM K4Fe[CN]6, 150 mM NaCl and 2 mM MgCl2 in PBS, pH 6.0] for 18 h at 37°C, rinsed with water and air‐dried. The local blue precipitate formed by cleavage of the substrate X‐Gal at pH 6.0 in senescent fibroblasts was assessed microscopically, and the positive cells were counted.

### 2.6 Western blot analysis

Thirty‐forty micrograms of lysate proteins for each sample together with the molecular weight Magic Mark (Invitrogen, Carlsbad, CA, USA) were subjected to 4–12% sodium dodecyl sulphate–polyacrylamide gel electrophoresis separation (Bis‐Tris Plus BOLT, Invitrogen) and transferred to polyvinylidene fluoride membranes (PVDF, Millipore, Burlington, MA, USA). The membranes were blocked in 5% skim milk and incubated overnight with the following specific primary antibodies: rabbit anti‐lamin B1 (Cell Signalling), mouse anti‐Tubulin (Sigma) followed by the suitable HRP‐conjugated secondary antibodies (Sigma‐Aldrich Chemicals). All resulting immunocomplexes were visualized with an enhanced chemiluminescence ECL detection system (GE Healthcare, Milano, Italy) and quantified by ImageJ software (NIH, Bethesda, MD, USA).

### 2.7 Confocal immunofluorescence

The primary antibodies used in immunofluorescence were rabbit anti‐γ‐H2Ax (Cell Signalling), mouse anti‐cGAS (Santa Cruz Biotechnology), rabbit anti‐lamin B1(Cell Signalling), rabbit anti‐NFkB (p65) (Cell Signalling) while the secondary were Cy3 or FITC–conjugated goat anti‐rabbit or mouse IgG (Sigma‐Aldrich Chemicals). DAPI were used for nucleus staining. The coverslips with the immune‐labelled cells were mounted with an anti‐fade mounting medium (Biomeda, Collegno, Italy) and analysed under a Bio‐Rad MRC 1024 ES confocal laser scanning microscope (Bio‐Rad) equipped with a 15‐mW Krypton/Argon laser source. The cells were observed with a Nikon Plan Apo X60 oil immersion objective (Nikon Instruments, Rome, Italy) at 595 nm. Series of optical sections (X‐ and Y‐steps: 512 × 512 pixels) were then obtained through the depth of the cells, with a thickness of 1 μm at intervals of 0.8 μm (Z‐step). A single composite image was obtained by superimposition of 20 optical sections for each sample. Total NFkB fluorescence intensity and Mander's coefficient (M1), used to assess NFκB p65 colocalization with the nucleus (DAPI), were determined by ImageJ software.

### 2.8 Measurement of DNA strand breaks and oxidized bases with the Comet assay

NHDFs were irradiated and treated with OLE and HT, as indicated in Materials and Methods section. After treatment, aliquots of the cells (50,000 cells in 50 μL) were resuspended in freezing medium (culture medium containing 40% FBS and 10% DMSO) and stored at −80°C. The frozen samples were de‐frosted and analysed for DNA damage by Comet assay as previously described.^23^ Briefly, each aliquot was rapidly melted in a 37°C water bath and immediately added with 167 μL of melted LMP agarose (LMA, 1% in PBS) kept at 37°C, to obtain a final 0.7% LMA concentration. Aliquots (70 μL) of this suspension were transferred onto clear microscopy slides pre‐coated with agarose and covered with 20 × 20 mm coverslips (2 duplicate gels per slide). Cells were lysed at 4°C for 1 h (lysis solution: NaCl 2.5 M, Na_2_EDTA 100 mM, Tris‐HCl 10 mM, TritonX‐100 1%, pH 10). The obtained nucleoids were then subjected to an alkaline unwinding step by incubating the slides for 20 min at 4°C in alkaline electrophoresis buffer (NaOH 300 mM, Na_2_EDTA 1 mM, pH 13). Following electrophoresis, slides were neutralized with two washes in 0.4 M Tris‐HCl, pH 7.4, briefly washed in distilled water and dried ON at RT. On the following day, gels were stained with SybrGold (Invitrogen, 1:10000 in Tris‐EDTA buffer) and left two days in the fridge. On the day of the microscopic analysis, images of the nucleoids were acquired and analysed using the Comet Assay IV image analysis system (Perceptive Instruments, UK) coupled to a Nikon Labophot‐2 epifluorescence microscope. Tail migration values (expressed as % tail DNA) were recorded for 100 randomly chosen cells for each experimental point and averaged. Each experiment was repeated three times, and the corresponding values were further averaged for each experimental point and expressed as mean ± SEM. For oxidatively generated damage detection, slides were washed with Enzyme Buffer (100 mM KCl, 0.5 mM EDTA, 40 mM HEPES and 0.2 mg/L BSA) 2 times for 5 min at 4°C, then incubated with 30 μL FPG enzyme per gel (crude E. Coli extract, Norgenotech, Oslo, Norway) or buffer only, for 45 min at 37°C. The DNA‐formamidopyrimidine glycosylase (Fpg) enzyme was used at 1:60.000 dilution. After this treatment, all slides proceeded to the unwinding step as described above. The net FPG sites, corresponding to the level of oxidized bases, were finally calculated by subtracting the % tail DNA value obtained in slides treated with enzyme buffer from that of the corresponding slides treated with the enzyme.

### 2.9 Preparation of conditioned media

DMEM plus 2% FBS was added, after washing with PBS, to NHDF cell cultures in a ratio of 1 mL/100,000 cells. Conditioned media were collected after 24 h incubation from untreated, 8 Gy‐treated, OLE and HT‐treated fibroblasts and centrifuged at 1500 rpm for 5 min. All media were stored at −80°C until use.

### 2.10 IL‐6 and IL‐8, CCL‐2/MCP‐1 and CCL‐5/RANTES detection in conditioned media

IL‐6 and IL‐8 were detected in cell‐conditioned media with Mini TMB ELISA Development Kits (PeproTech, DBA) according to the manufacturer's instructions, as previously described,^18^, ^20^ while CCL‐2/MCP‐1 and CCL‐5/RANTES were measured in cell‐conditioned media with pre‐coated ELISA kits (Peprotech, DBA) according to the manufacturer's instructions.

### 2.11 Statistical analysis

Statistical analyses of the data were performed with GraphPad Prism 9.0. The values are expressed as the mean ± standard error (SE). Two‐tailed unpaired Student's *t*‐test was performed for comparison of *n* = 2 groups. Comparisons of *n* > 2 groups were performed using one‐way ANOVA along with Bonferroni's post hoc test. For all statistical tests, *p* ≤0.05 were considered statistically significant and ≤0.01 very statistically significant.

## 3 RESULTS

### 3.1 Ionizing radiation (IR) induced premature senescence in NHDFs

**FIGURE 1:**
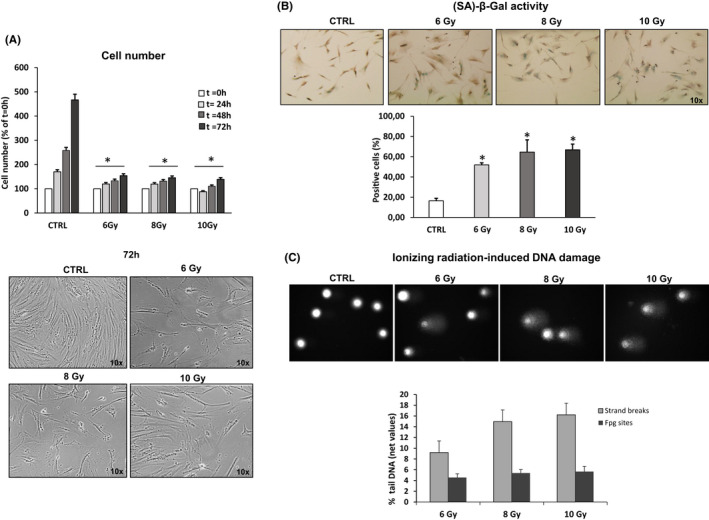
Ionizing radiation (IR)‐induced premature senescence in NHDFs. Confluent pre‐senescent NHDF cultures were exposed to different doses of ionizing radiation (6, 8 and 10 Gy) and mock irradiation was used as a control (CTRL). (A) Number of cells recovered at 24, 48 and 72 h after exposure at different doses of X‐rays. Histograms report the percentage of cell number respect to t = 0 h. Representative images of cell cultures photographed at 72 h post irradiation using a phase contrast microscope (100x magnification) were shown (bottom). Bars are the mean ± standard error (SE) of 3 experiments. *p < 0.05 vs. mock‐irradiated cells (CTRL). ANOVA multiple comparison test was performed. (B) Percentage of (SA)‐β‐Gal‐positive cells. Representative images of (SA)‐β‐Gal‐positive cells are reported (top, 100x magnification). *p < 0.05 vs. mock‐irradiated cells (CTRL) (C) DNA damage evaluation in irradiated NHDFs by Comet assay. Histograms report net radiation‐induced strand breaks and oxidized bases (Fpg sites), respectively (i.e. basal non‐irradiated value was subtracted), expressed as percent tail DNA (see Methods). Representative images of one Comet assay experiment are reported. Bars are the mean ± standard error (SE) of 3 experiments. *p < 0.05 vs. mock‐irradiated cells (CTRL)

Confluent pre‐senescent NHDF cultures were exposed to different doses of ionizing radiation (6, 8 and 10 Gy), and mock irradiation was used as a control (CTRL). To determine whether IR induced cellular senescence in NHDFs, we assessed three main parameters: the total number of cells collected and counted at 24, 48 and 72 h post irradiation to evaluate proliferation, (SA)‐β‐Gal activity and DNA damage evaluation. Figure 1A shows that the collected cell number was significantly decreased in NHDFs exposed to all doses of X‐ray compared with CTRL at any time of observation with approximately 30%, 50% and 70% reductions, respectively, indicating decreased proliferation capacity. In parallel, irradiated NHDFs showed a large, flattened morphology compared with non‐irradiated fibroblasts. In Figure 1B the induction of (SA)‐β‐Gal staining by IR is shown: in this case the 8 and 10 Gy doses appeared to be slightly more effective than 6 Gy. In fact, the number of (SA)‐β‐Gal‐positive cells resulted more than quadrupled with 8 and 10 Gy compared with CTRL. For DNA damage evaluation in irradiated NHDFs, we used the single‐cell gel electrophoresis method (Comet assay) to measure both strand breaks (SSBs) and oxidized bases. Irradiation with 8 Gy induced a higher level of DNA strand breaks compared with 6 Gy, whereas no further increase was observed with 10 Gy. As for DNA oxidative damage, no major difference was observed among the three irradiation conditions (Figure 1C). Based on these observations, we decided to perform all the experiments with 8 Gy irradiation.

### 3.2 Effects of OLE and HT treatment on stress‐induced premature senescence (SIPS) in NHDFs

**FIGURE 2:**
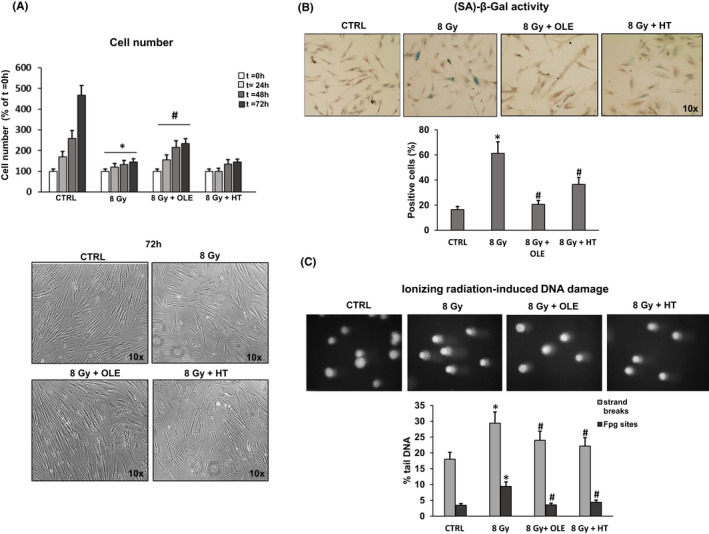
Effects of OLE and HT treatments on stress‐induced premature senescence (SIPS) in NHDFs. 24 h post irradiation, NHDFs were treated with 5 µM oleuropein aglycone (OLE) and 1 µM hydroxytyrosol (HT) for 2 weeks (5 treatments). (A) Number of cells recovered at 24, 48 and 72 h after the end of treatment. Histograms report the percentage of cell number respect to t = 0 h. Representative images of cell cultures photographed at 72 h using a phase contrast microscope (100x magnification) are shown. (B) Percentage of (SA)‐β‐Gal‐positive cells. Representative images of positive cells are reported (100x magnification). (C) DNA damage evaluation in untreated and OLE‐ or HT‐treated SIPS NHDFs by Comet assay. Histograms report strand breaks and oxidized bases (Fpg sites) expressed as percent tail DNA (see Methods). Representative images of one Comet assay experiment are reported. Bars are the mean ± standard error (SE) of 3 experiments. *p < 0.05 vs. mock‐irradiated cells (CTRL); #*p* < 0.05 vs. 8 Gy‐irradiated NHDFs

SIPS was induced in pre‐senescent NHDFs by 8 Gy, and mock irradiation was used as a control. 24 h post irradiation, NHDFs were treated with 5 µM oleuropein aglycone (OLE) and 1 µM hydroxytyrosol (HT), as described in Material and Methods section. To investigate the effect of treatment on the SIPS‐phenotype, we evaluated the total number of cells collected and counted at the end of the treatment, (SA)‐β‐Gal activity, and DNA damage in pre‐senescent (CTRL), untreated (8 Gy) or OLE‐ and HT‐treated SIPS NHDFs. In Figure 2A, a significant reduction in cell number is shown along with a more than quadrupled number of (SA)‐β‐Gal‐positive cells (Figure 2B) in 8 Gy‐NHDFs compared with untreated cells, confirming the efficacy of 8 Gy dose in inducing premature senescence. OLE brought about a significant increment in cell number with approximately 50% increases at 48 and 72 h, while HT did not produce any effect compared to irradiated cells. As for (SA)‐β‐Gal staining, both phenol treatments were effective in reducing the percentage of positive cells compared to SIPS NHDFs, with 70% and 40% reductions respectively. For DNA damage evaluation with the Comet assay, a significant protective effect of OLE and HT was observed towards the SIPS‐associated increase in DNA strand breaks (SSBs) and in oxidative damage (measured as FPG‐sensitive sites), which were lowered by the treatment to the level of non‐irradiated NHDFs (Figure 2C).

### 3.3 IR induces lamin B1 loss, CCFs accumulation and cGAS‐STING pathway activation in SIPS NHDF

To further analyse the effect of ionizing radiation on DNA damage, we first examined the status of nuclear DNA and proteins.

**FIGURE 3:**
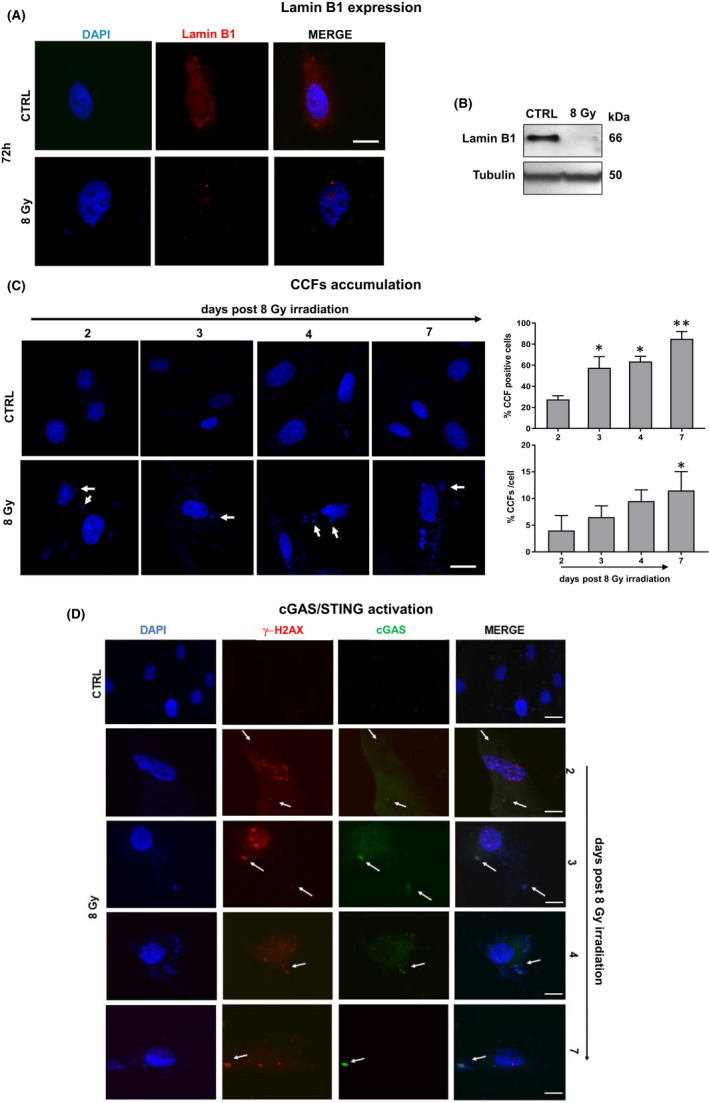
Lamin B1 expression, CCFs accumulation and cGAS‐STING pathway activation in SIPS NHDFs. (A) Confocal analysis of lamin B1 expression in 8 Gy‐irradiated NHDFs. Scale bar =20 µm. (B) Western Blotting analysis of lamin B1 in 8 Gy‐irradiated NHDFs. Tubulin was used as loading control. (C) CCF release in SIPS NHDFs by confocal immunofluorescence analysis. Histograms show the percentage of CCF‐positive cells in 8 Gy‐irradiated compare to mock‐irradiated cells (CTRL) and the percentage of CCFs per cell, respectively, at 2, 3, 4 and 7 days post irradiation. Data represent the mean ± standard error (SE) of 3 experiments. ANOVA multiple comparison test was performed. *p < 0.05 vs. mock‐irradiated cells (CTRL). **p < 0.01 vs. mock‐irradiated cells (CTRL). (D) γ‐H_2_Ax and cGAS‐STING pathway activation in SIPS NHDFs by confocal immunofluorescence analysis at 2, 3, 4 and 7 days post irradiation. CCFs are indicated by the arrows. Scale bar =20 µm

As the loss of the nuclear lamina protein, lamin B1, is responsible for a compromised integrity of the nuclear envelope contributing to the formation of cytoplasmic chromatin fragments (CCFs), we analysed lamin B1 expression by immunofluorescence. As shown in Figure 3A, 8 Gy‐irradiated fibroblasts showed lack of expression of this nuclear protein, which was instead detected in non‐irradiated cells (CTRL). This data was confirmed by Western Blotting analysis (Figure 3B).

Figure 3C shows that damaged chromatin was released from the nucleus to cytoplasm, forming DNA‐containing blebs known as CCFs in SIPS fibroblasts. In particular, the percentage of CCF‐positive cells increased with senescence progressing (from 2 to 7 days post irradiation), being significantly different (*p *≤ 0.05) already after 3 days post irradiation and very significantly different (*p *≤ 0.01) at 7 days post irradiation, reaching at this time almost 100% of positive cells in 8 Gy‐irradiated compared to non‐irradiated cells (CTRL). In addition, the level of cytosolic dsDNA was also increased in senescent cells. Indeed, we observed an increment (from 2 to 7 days post irradiation) in the number of DNA‐containing blebs/cell, which was significantly different in 8 Gy‐irradiated compared to non‐irradiated fibroblasts at 7 days post irradiation. CCFs contain genomic DNA, γ‐H2AX, and heterochromatin markers H3K9me3 and are recognized by the cytosolic DNA sensor cGAS. Therefore, we examined the immunofluorescence co‐staining of γ‐H2AX and cGAS in CCFs, and found colocalization of γ‐H2AX and cGAS in DAPI‐stained CCFs released by 8 Gy‐irradiated NHDFs (Figure 3D). Taken together, these data indicate that ionizing radiation induces CCFs accumulation and cGAS/STING pathway activation in SIPS NHDFs.

### 3.4 Effects of OLE and HT treatments on DNA damage and nuclear leakage in SIPS NHDFs

**FIGURE 4:**
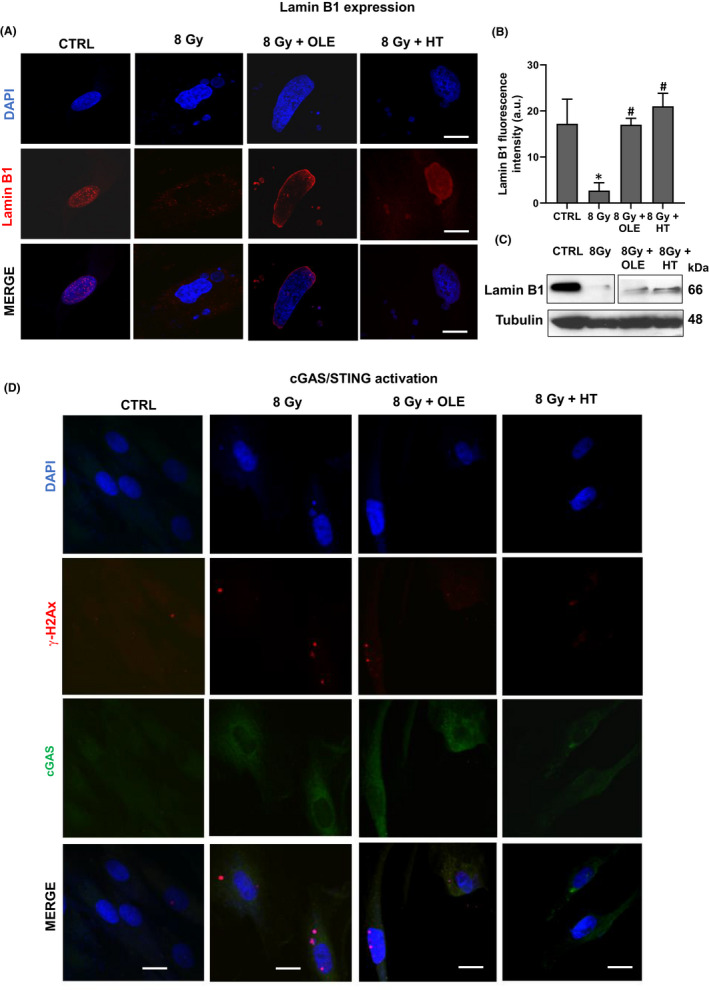
Effects of OLE and HT treatment on lamin B1 expression and cGAS‐STING pathway activation in SIPS NHDFs. (A) Confocal analysis of lamin B1 expression in OLE‐ and HT‐treated SIPS NHDFs. Representative images were showed. Scale bar =20 µm. (B) Histogram shows the mean of lamin B1 fluorescence intensity measured on 40 cells. *p < 0.05 vs. mock‐irradiated cells (CTRL). #*p* < 0.05 vs. 8 Gy‐irradiated cells. (C) Western Blotting analysis of lamin B1 in OLE‐ and HT‐treated NHDFs. The clear space between the bands of ctrl and 8 Gy and the bands of 8 Gy + OLE and 8 Gy + HT, indicative of splicing, exists because, originally, we also evaluated lamin B1 expression in another sample not providing valuable information for the interpretation of our data. Tubulin was used as loading control. (D) Confocal analysis of cGAS‐STING pathway activation in OLE‐ and HT‐treated SIPS NHDFs. Scale bar =20 µm

To study the effect of OLE and HT treatment on DNA damage and nuclear leakage in SIPS NHDFs, we analysed the markers lamin B1 expression, CCFs release, γ‐H2AX expression and cGAS/STING pathway activation, in untreated or OLE‐ and HT‐treated SIPS NHDF. As shown in Figure 4A and B OLE and HT treatments preserved lamin B1 expression in irradiated cells compared to untreated senescent cells in which the expression of this nuclear protein was completely lost. This data was confirmed by Western blotting analysis (Figure 4C). Consequently, a reduced release of CCFs was observed in SIPS NHDFs treated with olive phenols compared to untreated cells both in terms of positive cell number and of DNA‐blebs number per cell. We then analysed the effect of OLE and HT treatment on γ‐H2AX expression and cGAS/STING pathway activation and observed a reduced intensity of γ‐H2AX and cGAS in OLE‐ and HT‐treated SIPS NHDF compared to untreated senescent cells. In particular, HT‐treated fibroblasts showed a complete loss of γ‐H2AX and cGAS expression (Figure 4D). These observations suggest that OLE and HT reduced DNA damage and preserved the integrity of the nuclear envelope, preventing the shedding of damaged chromatin from the nucleus to the cytoplasm.

### 3.5 Effects of OLE and HT treatment on NFkB activation and SASP induction in SIPS NHDFs

**FIGURE 5:**
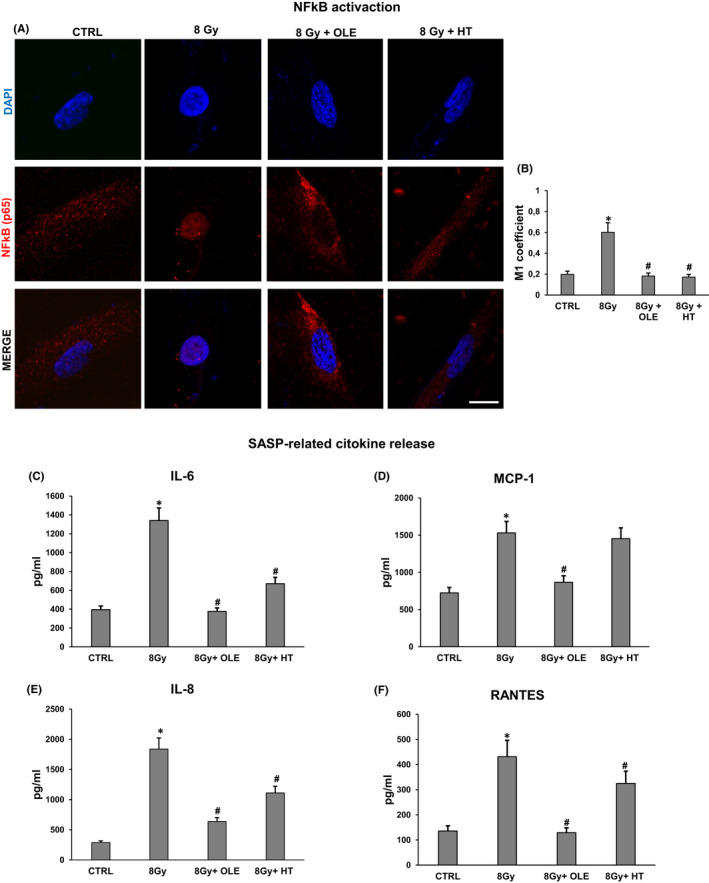
Effects of OLE and HT treatment on NFkB activation and SASP factors release in SIPS NHDFs. (A) NFkB intracellular localization analysed by confocal immunofluorescence. Scale bar =20 µm. (B) Histogram show NFkB fluorescence nuclear localization (NFkB/DAPI) by Mander's coefficient (M1) using Image J software. (C) IL‐6 (D) MCP‐1 (E) IL‐8 and (F) RANTES quantification in conditioned media collected from OLE‐ and HT‐treated SIPS NHDFs by ELISA assay. Histograms report pg/ml of the selected cytokines. Bars are the mean ± standard error (SE) of 3 experiments. *p < 0.05 vs. mock‐irradiated cells (CTRL); #*p* < 0.05 vs. 8 Gy‐irradiated NHDFs

We then addressed the question whether OLE and HT were able to also reduce the SASP. As cGAS‐STING signalling pathway promotes in senescent cells a SASP programme through NFkB activation, we examined the effect of OLE and HT treatments on NFkB nuclear localization by immunofluorescence. Figure 5A shows that irradiation induced an increment in NFkB nuclear localization, measured by Mander's coefficient M1, that was lowered to the control levels by both olive phenols treatments, being HT slightly more effective than OLE (Figure 5B). Consequently, we analysed the expression level of IL‐6, IL‐8, MCP‐1 and RANTES, well‐known SASP cytokines released by senescent fibroblasts.^24^, ^25^ As shown in Figure 5, panels C, D, E and F, a reduction of the SASP markers IL‐6 and IL‐8, MCP‐1 and RANTES release in extracellular medium was observed in SIPS NHDFs after treatment with OLE and HT (80% and 50%, respectively, for IL‐6, 70% and 40% for IL‐8, 44% and 6% for MCP‐1, 70% and 25% for RANTES) confirming that both treatments are efficient in modulating the ionizing radiation‐induced SASP in NHDFs.

## 4 DISCUSSION

Accumulating evidence indicates that olive phenols exert a potent antioxidant activity associated with anti‐inflammatory and anti‐tumour properties.^26^, ^27^, ^28^ The present study shows that deglycosylated product of oleuropein and hydroxytyrosol, two of the most abundant phenols in olive fruit and derivatives, can exert protective effects on senescence‐associated DNA damage, increasing nuclear DNA stability and attenuating the senescence‐associated inflammatory phenotype in NHDFs. Here, we developed a model of irradiation‐induced premature senescence, the so‐called SIPS. This form of senescence can be induced by diverse stimuli.^29^ For example, the exposure to DNA damaging agents such as ionizing radiation and various anti‐cancer chemotherapy agents are known to give rise to a senescent phenotype in both normal and cancer cells, which is often termed ‘Therapy‐Induced Senescence’ (TIS).^30^ Inducing cancer cell senescence by chemotherapy and radiotherapy may provide an appealing option to limit cancer progression and to improve the prognosis of various solid and haematological malignancies. However, in recent years, several studies have highlighted that the acquisition of a senescent phenotype by surrounding stromal cells could have detrimental effects, causing the generation of a SASP‐mediated chronic inflammatory and tumour‐permissive microenvironment, contributing to therapeutic resistance, malignant cell spreading and cancer relapse.^25^ Therefore, senescence induction can be considered as a ‘double‐edged sword’. On one hand, cellular senescence might limit the propagation of damaged cells and malignant transformation. On the other hand, senescent cells might induce a pro‐tumoral microenvironment and support a cell proliferative reprogramming. In addition, recent works demonstrated an early protective role of SASP‐evoked immune response promoting innate and adaptive immune cells recruitment to tumour cells and pre‐malignant lesions.^31^

Based on these considerations, we developed a model of stress‐induced premature senescence exposing confluent pre‐senescent NHDF cultures to different doses of ionizing radiation (6, 8 and 10 Gy). Then, we demonstrated that ionizing irradiation and, in particular 8 Gy dose, was effective in inducing premature senescence assessing three main parameters: the total number of cells collected and counted at 24, 48 and 72 h post irradiation, (SA)‐β‐Gal activity and DNA damage evaluation. After validation of our model of senescence, we treated SIPS NHDFs continuously with 5 µM OLE or 1 µM HT for 2 weeks (5 treatments). We showed that the treatment with OLE was able to increase the recovered cell number and that both treatments induce a significant decrease of the senescence marker (SA)‐β‐Gal. These data agree with previous observations in pre‐senescent human lung and neonatal human dermal fibroblasts treated with HT and oleuropein, where a reduction of senescent and (SA)‐β‐Gal‐positive cells number as well as p62 protein expression was observed.^17^ In a recent study, it was found out that human embryonic fibroblasts IMR90 and W138 cells treated with oleuropein showed a life span extended by approximately of 15% and a delay in the appearance of senescence morphology.^32^ Taken together, these results suggest that olive phenols can modulate cellular senescence, the negative side effects of SASP thereby affecting ageing process.

As for DNA damage, evaluated by Comet assay, both polyphenols determined a significant protective effect towards the senescence‐associated increase in DNA strand breaks and oxidative damage.

We thus hypothesized that an increased nuclear DNA stability might lead to reduced CCF release in the cytoplasm and, in turn, to reduced inflammatory signalling from cytoplasmic DNA‐sensing pathways. Here, for the first time, we report that both treatments were able to preserve lamin B1 expression, which resulted otherwise completely lost in 8 Gy‐irradiated NHDFs. Lamin B1 is one of the nuclear lamins (B1, B2, A and C) that compose the nuclear lamina and serve to maintain nuclear stability and chromatin organization.^33^ Downregulation of lamin B1 in senescent cell is a trigger of CCFs formation.^34^ Recent studies showed that CCF formation in IR‐induced senescent IMR90 cells may be also triggered by dysfunctional mitochondria that activate a ROS‐c‐Jun N‐terminal kinase (JNK) retrograde and an inflammatory signalling pathway.^35^ These pathways could be targetable by molecules such as polyphenols with proven anti‐inflammatory, antioxidant and autophagy–mitophagy activity. In this context, the olive oil polyphenols effect we observed on reducing CCF formation could also arise from the protective function of these molecules in modulating inflammatory and ROS‐dependent signalling pathways.

Most of these effects have been associated to the ability of EVOO polyphenols to control cell signalling pathways and to modulate the expression of pro‐ and anti‐apoptotic and anti‐autophagic factors, also through epigenetic modifications, counteracting oxidative, environmental stress, proteotoxicity, and cascade reactions responsible of chronic age‐related diseases. The health effects elicited by these plant products could be discussed also in the light of the hormesis theory, an evolutionarily conserved biphasic dose‐response that induce beneficial effects to a biological system or the whole organism.^36^, ^37^ Recently, in vivo studies suggested that polyphenols likely counteract the effects of inflammatory stimuli by acting as modulators of stress responsive mechanisms, which result in adaptive stress resistance. The mechanistic profile can be traced back to the activation of the Nrf2/ARE pathway that induces the upregulation of *vitagenes*, a group of *genes* involved in preserving cellular homeostasis during stressful conditions and that encode for γ‐glutamyl cysteine synthetase (γ‐GCS), HO‐1, heat‐shock protein 70 (Hsp70), thioredoxin and sirtuin‐1 (Sirt1). Moreover, Nrf2 activation inhibits the pro‐inflammatory mediators such as cytokines, COX‐2 and iNOS production that are responsible for the increase in oxidative and nitrosative stress. Reactive nitrogen species (RNS) have been claimed to play a crucial role in many different processes, both physiological such as neuromodulation, synaptic plasticity, macrophage‐mediated immunity, cardiovascular tonicity and cell proliferation and pathological including ischemia, cancer and age‐related disorders.^38^, ^39^ In particular, a large body of evidence suggested a correlation between the mechanism of nitrosative stress and HSP induction.^40^

In addition, RNS play important role in different SASP signalling pathways causing DNA damage that can lead to protein kinase ataxia‐telangiectasia‐mutated protein (ATM) and Chk2 activation, or NF‐κB activation and IL‐6/IL‐8 gene transcription. All these changes can induce the initiation of cellular senescence programmes. EVOO polyphenols, through the activation of hormetic pathways, including *vitagenes*, contribute to the redox balance of the cell, with ensuing upregulation of cell antioxidant defences.

Considering the pivotal role of oxidative and nitrosative stress in DNA damage, manipulation of ROS and RNS cellular content by EVOO polyphenols may represent a promising treatment option to preserve the protein homeostasis and cellular redox balance and reduce damage in various pathological states.^39^, ^41^, ^42^ Moreover, polyphenols could contribute to maintenance of optimal long‐term health conditions by a complex network of longevity assurance processes that are controlled by *vitagenes* and prevent CCF formation and senescence‐associated inflammation in our cell system model.

Cytoplasmic DNA, typically a hallmark of pathogen infection, activates the cytosolic DNA‐sensing cGAS‐STING pathway that in turn drives the type I interferon (IFN) pathway and pro‐inflammatory cytokine production to induce the innate immune response against invading pathogens.^43^ However, CCFs and the cGAS‐STING pathway also induces the SASP in all forms of senescence.^14^, ^35^ CCFs are recognized by the DNA sensor cGAS, which converts adenosine 5' triphosphate (ATP) and guanosine 5' triphosphate (GTP) into cyclic GMP‐AMP (cGAMP) upon DNA binding.^44^ With confocal microscopy analysis we showed that OLE and HT treatment induced a reduction of γ‐H2AX expression and cGAS/STING pathway activation. At the same time, we observed a reduction of NFkB activation, as indicated by nuclear localization, in OLE‐ and HT‐treated fibroblasts.

Finally, we analysed the effect of olive phenols on ionizing radiation‐mediated SASP. We measured in conditioned media collected from OLE‐ and HT‐treated and untreated SIPS NHDFs the level of the pro‐inflammatory cytokines IL‐6, IL‐8, CCL‐2/MCP‐1 and CCL‐5/RANTES. For all the analysed cytokines, we found an increased release in irradiated‐NHDF extracellular media that resulted lowered in the media collected from phenol‐treated SIPS NHDFs, being OLE more effective than HT. In addition to the classical SASP cytokines, IL‐6 and IL‐8, previously showed to be associated with the secretory phonotype in a model of replicative senescence,^19^, ^21^ we have observed, for the first time, a modulation by OLE and HT treatment of chemokine ligand 2, also known as Monocyte Chemoattractant Protein 1 CCL‐2/MCP‐1 and chemokine ligand 5 (also known as RANTES), two important SASP‐associated chemokines involved in the leucocyte recruitment. The MCP‐1 is a chemokine that recruits immune cells to inflammatory sites through binding to C‐C chemokine receptor type 2 (CCR2).^45^ High levels of circulating MCP‐1 are common in aged individuals, and in vitro they appear to confer senescence even to neighbouring normal cells in an autocrine and paracrine fashion.^46^, ^47^, ^48^ The RANTES is a small, 8 kDa cytokine that attracts T cells, macrophages, endothelial cells and fibroblasts through binding to receptors CCRs.^49^ In addition to its role in chemotaxis in the immune system, RANTES is involved in most age‐related conditions, such as pulmonary hypertension,^50^ type 2 diabetes^51^ and kidney ageing.^52^ OLE and HT have been reported to have antioxidant and anti‐inflammatory effect due to their ability to inhibit the synthesis of pro‐inflammatory mediators such as IL‐1β, TNF‐α, IL‐10, COX‐2, iNOS, TGF‐β1, MCP‐1 and NFkB in different models of inflammation.^53^, ^54^, ^55^, ^56^ Here, we demonstrated the ability of OLE and HT to modulate the senescence‐associated secretion of MCP‐1 and RANTES in irradiated NHDFs.

In conclusion, our results demonstrate a novel mechanism of nuclear damage protection by OLE and HT which is critical for the modulation of the potentially deleterious growth‐promoting and pro‐tumoral effect of the senescent cell secretome. Furthermore, these findings indicate that molecules such as oleuropein and hydroxytyrosol can modulate irradiation‐induced SASP in normal cells, indicating the possibility to reduce undesired effects of radiation therapy in non‐tumour cells. At the same time, our observations pave the way for future studies aimed at evaluating the effect of these molecules on the senescence of cancer cells themselves. Small drug‐like molecules such as olive phenols, able to modulate normal cell SASP without abrogating it, can thus contribute to the safe‐implementation of therapeutic strategies to fight cancer and age‐related pathologies.

## CONFLICT OF INTEREST

None declared.

## AUTHOR CONTRIBUTIONS

**Elena Frediani:** Data curation (equal); Formal analysis (equal); Investigation (equal); Methodology (equal). **Francesca Scavone:** Data curation (equal); Formal analysis (equal); Investigation (equal); Methodology (equal). **Anna Laurenzana:** Data curation (equal); Formal analysis (equal); Methodology (equal); Resources (equal); Supervision (equal); Validation (equal). **Anastasia Chillà:** Data curation (equal); Formal analysis (equal); Methodology (equal); Resources (equal); Supervision (equal); Validation (equal). **Katia Tortora:** Data curation (equal); Formal analysis (equal). **Ilaria Cimmino:** Data curation (equal); Formal analysis (equal). **Manuela Leri:** Data curation (equal); Formal analysis (equal); Methodology (equal); Resources (equal). **Monica Bucciantini:** Conceptualization (equal); Writing – original draft (equal). **Monica Mangoni:** Data curation (equal); Formal analysis (equal); Methodology (equal); Resources (equal). **Gabriella Fibbi:** Conceptualization (equal); Supervision (equal); Validation (equal). **Mario Del Rosso:** Conceptualization (equal); Writing – original draft (equal). **Alessandra Mocali:** Conceptualization (equal); Supervision (equal); Validation (equal). **Lisa Giovannelli:** Conceptualization (equal); Data curation (equal); Formal analysis (equal); Writing – original draft (equal). **Francesca Margheri:** Conceptualization (equal); Data curation (equal); Formal analysis (equal); Methodology (equal); Supervision (equal); Validation (equal); Writing – original draft (equal).

## CONSENT TO PUBLISH

All of the authors have consented to publish this research.

## Data Availability

The data used to support the findings of this study are available from the corresponding author upon request.
